# The impact of cannabis use on patients enrolled in opioid agonist therapy in Ontario, Canada

**DOI:** 10.1371/journal.pone.0187633

**Published:** 2017-11-08

**Authors:** Alexandra M. Franklyn, Joseph K. Eibl, Graham J. Gauthier, David C. Marsh

**Affiliations:** 1 Northern Ontario School of Medicine, Sudbury, Ontario, Canada; 2 Canadian Addiction Treatment Centers, Richmond Hill, Ontario, Canada; Waseda University, JAPAN

## Abstract

**Background:**

With the Canadian government legalizing cannabis in the year 2018, the potential harms to certain populations—including those with opioid use disorder—must be investigated. Cannabis is one of the most commonly used substances by patients who are engaged in medication-assisted treatment for opioid use disorder, the effects of which are largely unknown. In this study, we examine the impact of baseline and ongoing cannabis use, and whether these are impacted differentially by gender.

**Methods:**

We conducted a retrospective cohort study using anonymized electronic medical records from 58 clinics offering opioid agonist therapy in Ontario, Canada. One-year treatment retention was the primary outcome of interest and was measured for patients who did and did not have a cannabis positive urine sample in their first month of treatment, and as a function of the proportion of cannabis-positive urine samples throughout treatment.

**Results:**

Our cohort consisted of 644 patients, 328 of which were considered baseline cannabis users and 256 considered heavy users. Patients with baseline cannabis use and heavy cannabis use were at increased risk of dropout (38.9% and 48.1%, respectively). When evaluating these trends by gender, only female baseline users and male heavy users are at increased risk of premature dropout.

**Interpretation:**

Both baseline and heavy cannabis use are predictive of decreased treatment retention, and differences do exist between genders. With cannabis being legalized in the near future, physicians should closely monitor cannabis-using patients and provide education surrounding the potential harms of using cannabis while receiving treatment for opioid use disorder.

## Introduction

Cannabis is a plant that contains a psychotropic chemical known as delta-9-tetrahydrocannabinol (THC)[1]. Cannabis is widely used in the general population with 3.6 million Canadians—12% of the population—reporting past-year cannabis use[2]. Importantly, one third of past-year cannabis users report using it on a daily or almost daily basis[2]. The median age of initial cannabis use in Canada is only 17, with 21% of youth aged 15 to 19 reporting past-year use[2].

Many studies have found a public health benefit from the legalization of cannabis. Both Canada and the U.S. have recently modified legislation with regards to medical use of cannabis, particularly for its therapeutic role[3]; these changes appear to have had a positive effect, particularly on patients with opioid use disorder (OUD). In the U.S., states with medical cannabis laws have 25% lower opioid overdose mortality rates[4]. Additionally, a retrospective cross-sectional study found that patients who use cannabis to manage chronic pain experience 64% less opioid use and have increased quality of life[5]. Studies have found other public health benefits from legalization of cannabis, including a decrease in cannabis-related arrests[6].

Although there is evidence to support that cannabis legalization will benefit the population as a whole, cannabis legalization remains a controversial topic. Despite being regarded by many as being harmless[7], studies have found that cannabis is harmful to the adolescent brain, that it impairs cognition, lowers life satisfaction, is correlated with psychotic episodes and disorders[8], and is associated with poly-substance use[9, 10]. With Canada having passed federal legislation that will lead to the legalization of cannabis for recreational use[11], there are many questions that remain unanswered. For example, there is currently debate with regards to whether legalization of cannabis will lead to increased consumption, with some studies suggesting cannabis use in the general population will increase[12–14], and others suggesting it will not[15].

Despite findings that suggest cannabis legalization will have positive outcomes overall, the potential harms of cannabis legalization must be closely monitored, as there may be certain populations who are at increased risk of suffering cannabis-related harms. One such population is those patients with OUD. With the current opioid crisis, it is important to better understand the implications of cannabis use and its relationship to opioid use, particularly its impact for those patients receiving treatment for OUD.

Opioids are a family of drugs that are central nervous system depressants. For those patients who develop OUD, there is a treatment available known as opioid agonist therapy (OAT). OAT is a maintenance therapy whereby the patient is relieved of their opioid withdrawal and is able to return to their previous functioning. Across Canada, OAT is currently available in the form of methadone and buprenorphine/naloxone, and in the form of injectable heroin or hydromorphone treatment in one site in British Columbia. Studies have found that patients who are receiving treatment for OUD have a much higher prevalence of cannabis use than the general population[16], with cannabis being the most commonly used drug among patients receiving OAT[17, 18]. While concurrent drug use in OAT is generally associated with poorer treatment outcomes[19–22], the literature is mixed as to the impact of cannabis on OAT outcomes, with some studies finding cannabis use predictive of poorer treatment outcomes[23, 24] and others finding no negative impact [25, 26]. With pending legalization, it is important that the impact of cannabis use on OAT outcomes is better understood to ensure that patient safety is maintained to maximize positive treatment outcomes, including treatment retention.

It would appear that much remains unanswered about the impact of cannabis use on the ever-growing opioid-using population. With the Canadian Federal Government aiming to legalize cannabis in the year 2018[27], it is critical that the potential risks and harms are studied prior to legalization in order to understand whether this population requires extra monitoring post-legalization. In this paper, we characterize the impact of cannabis use on OAT treatment retention for patients with OUD in Ontario, Canada.

## Methods

### Clinical context

In Ontario, OAT is regulated by formal treatment guidelines established by the College of Physicians and Surgeons of Ontario (CPSO), which set out expectations with respect to physician practice and are enforced through peer-audits[28]. These guidelines are in addition to the federal requirement for an exemption to prescribe methadone. Variability of practice within the guidelines is possible, but is generally limited. Ontario has a single payer healthcare system, whereby all residents have equal access to OAT through the Ontario Health Insurance Plan (OHIP).

This study is based on the electronic medical records of patients treated within the Ontario Addiction Treatment Centres (OATC), a network of 58 OAT clinics across the Province of Ontario that are operated under common management. Standardized evidence-based best practice policies and operating procedures are in place within the clinic network, which further limit the likelihood of variability of treatment between sites and physicians. To maintain consistency, patients are typically seen by the same physician throughout the course of their treatment.

### Cohort definition

We conducted a retrospective cohort study of patients initiating OAT within the OATC network for the first time between January 1^st^, 2011 and June 17^th^, 2012 in the Province of Ontario. We defined first time OAT as no previous history of methadone or buprenorphine use in the OATC network, based on review of records dating back to 1999. Patients were started on methadone and were allowed to transition to buprenorphine/naloxone over the course of treatment. Patients with less than five urine tests for THC were excluded from analysis. Patients were at least 15 years or older (patients <18 years of age accounted for < 1% of cohort), and were residents of Ontario. All patients were followed from the date of OAT initiation to the date of medication discontinuation, or end of the study period (June 2013). Drug discontinuation was defined as a patient not receiving a dose of methadone or buprenorphine/naloxone for 30 consecutive days.

### Data sources

The dataset used for this study was derived from anonymized electronic medical records from the OATC network of 58 addiction treatment centers across the Province of Ontario. Methadone prescribing, treatment delivery, and data management are harmonized across the clinic network. Prior to data analysis, personal identifiers were replaced with an encrypted unique identifier. Cluster analysis (testing relation between individual clinic and treatment retention) did not reveal any significant differences among clinics with respect to increased/decreased treatment retention by individual clinic or physician.

### Variables

The following variables were studied: geographic location (North vs. South, rural vs. urban), age, gender, and first-month cannabis use. Patients were considered residents of Northern Ontario or Southern Ontario defined by the Local Health Integration Network (LHIN). If patients lived in LHIN 13 or 14, they were considered residents of Northern Ontario. Rurality was defined according to the Rurality Index for Ontario (RIO), where a score of 40 or higher is rural. Patients were considered to be baseline cannabis users if they had a least one cannabis-positive urine sample in the first month of treatment.

### Cannabis use

Patients were categorized by baseline and ongoing cannabis use based on urine immunoassay testing, which has the ability to detect THC[29]. Regular cannabis use can be detected for up to 28 days, whereas occasional use can be detected for up to 3–4 days[29]. Patients were also screened for their OAT metabolite to ensure patients were not diverting their prescribed medication. Patients were considered to be baseline cannabis users if they had any THC-positive urine samples in their first month of treatment. Patients were also stratified into two groups depending on the proportion of cannabis-positive urine samples throughout treatment: [0–75%) and [75–100%], with square brackets implying inclusivity and round brackets implying exclusivity. Patients were considered heavy users if 75% or more of their urines were cannabis-positive and were considered non-heavy users if less than 75% of their urines were cannabis-positive.

### Definition of treatment retention

Patients were followed from treatment initiation for at least one year, to a maximum follow-up date of June 17^th^, 2013. For the purpose of this study, treatment retention has been defined as being in treatment for one year of continuous and uninterrupted OAT, based on having received a prescription refill (for methadone or buprenorphine/naloxone) within 30 days of the previous prescription end date (i.e. no period of 30 consecutive days without a dose of medication).

### Statistical analysis

Descriptive statistics were summarized for baseline characteristics of patients, and standardized differences were used to compare patient groups. Baseline characteristics included percentage of patients that were male/female, Northern/Southern, and rural/urban, median age, median peak methadone dose, median days retained, the percentage of cannabis-positive urine samples, 12-month cannabis use, and the one-year retention rate. For the purpose of this study, only a patient’s first treatment episode was considered. For the primary analysis, a Cox proportional hazard analysis was used to characterize the time to treatment discontinuation between the cannabis-positive and -negative patient groups with adjustment for the impact of age, gender, first-month cannabis use, and Northern and rural location. Cox Proportional analysis was performed using SPSS 24. Age, gender, and Northern and rural location were analysed with Logistic regression to characterize first-month cannabis use.

## Results

### Patient demographics

Our cohort consisted of 644 patients across 58 clinics. Characteristics of baseline cannabis users and non-users can be found in Table 1. The median age was 31 years and 59.6% of the cohort was male. 28.4% of the population resided in Northern Ontario (where 20 of the 58 clinics were located) and only 9.2% resided in a rural community.

**Table 1:** Characteristics of baseline cannabis users and non-users.

		Initially Negative (n = 316, 50.1%)	Initially Positive (n = 328, 49.9%)
**Male / Female**		178 (56.3%) / 138 (43.7%)	206 (62.8%) / 122 (37.2%)
**South / North**		235 (74.4%) / 81 (25.6%)	226 (68.9%) / 102 (31.1%)
**Urban / Rural**		290 (91.8%) / 26 (8.2%)	295 (89.9%) / 33 (10.1%)
Median Age (Q_1_, Q_3_; SD)		33 (26, 44; SD = 11)	29 (24, 37; SD = 10)
**Median Peak Methadone Dose (Q_1_, Q_3_; SD)**		90 (65, 110; SD = 33)	80 (60, 105; SD = 32)
**Median Days Retained (Q_1_, Q_3_; SD)**		444 (349, 554; SD = 167)	405 (261, 515; SD = 162)
Median Percent Positive Results (Q_1_, Q_3_; SD)		0.0 (0.0, 13.4; SD = 24.1)	88.9 (69.6, 100.0; SD = 24.7)
**Percent Positive Results**	[0, 25)	253 (80.1%)	18 (5.5%)
	[25, 50)	23 (7.3%)	24 (7.3%)
	[50, 75)	21 (6.6%)	49 (14.9%)
	[75, 100]	19 (6.0%)	237 (72.3%)
**Not-Retained / Retained** Day 365		92 (29.1%) / 224 (70.9%)	128 (39.0%) / 200 (61.0%)

### Baseline cannabis use

Of the 644 patients, 328 patients (50.9%) had at least one cannabis-positive urine in the first month of treatment, and 316 (49.1%) did not. The baseline positive group had a higher proportion of males (62.8% vs. 56.3%), a higher proportion of rural residents (10.1% vs. 8.2%), and had a lower median age (29 vs. 33). There were no significant associations between being a baseline cannabis user and gender or geographic location. First-month cannabis users had a decreased median peak dose of methadone (80 mg vs. 90 mg), and had a lower median retention of 405 days, compared to 444 days. Overall, first-month cannabis users had a lower one-year retention rate of 61.0% compared to 70.9%.

### Retention and baseline cannabis use

**Fig 1:**
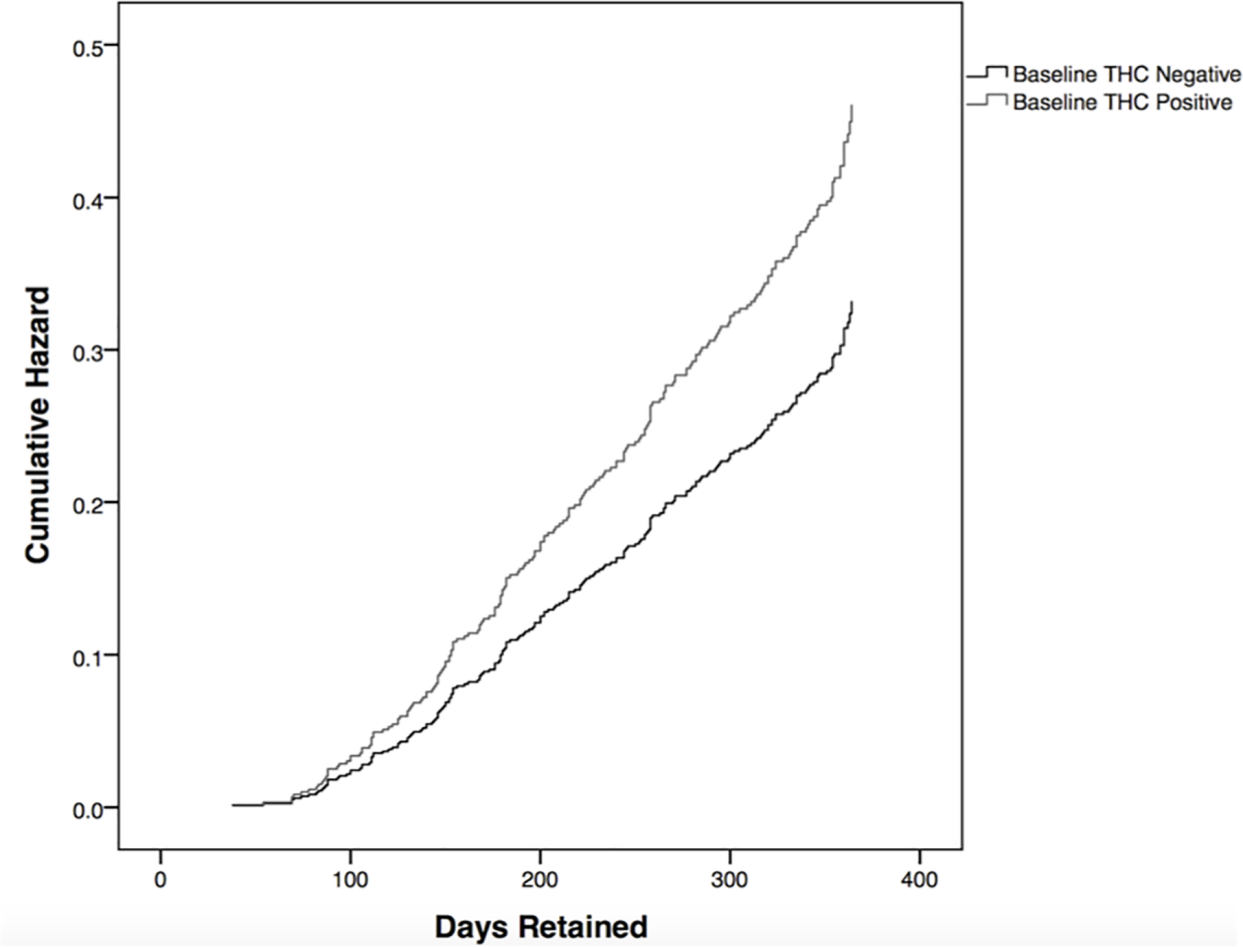
Likelihood of treatment dropout by baseline cannabis use. A Cox proportional hazard analysis was used to characterize the time to treatment discontinuation between the patient groups. Baseline cannabis users were 38.9% more likely to drop out of treatment than baseline non-users [_a_HR = 1.389 (95% CI 1.0573–1.83)].

Baseline cannabis users were 38.9% more likely to drop out of treatment than baseline non-users [_a_HR = 1.389 (95% CI 1.0573–1.83)] (Fig 1). The variables included in the analysis were: gender (female [_a_HR = 0.69 (95% CI 0.517–0.923)]), geography (North [_a_HR = 0.487 (95% CI 0.335–0.707)] and rural [_a_HR = 0.947 (95% CI 0.545–1.646)], and first-month cannabis use [_a_HR = 1.389 (95% CI 1.057–1.825)]. Of those patients who did not have cannabis-positive urine samples in their first month of treatment, 70.9% were retained at one year; this was compared to 61.0% for patients who were positive at baseline (Table 1).

### Proportion of cannabis-positive urine samples

**Fig 2:**
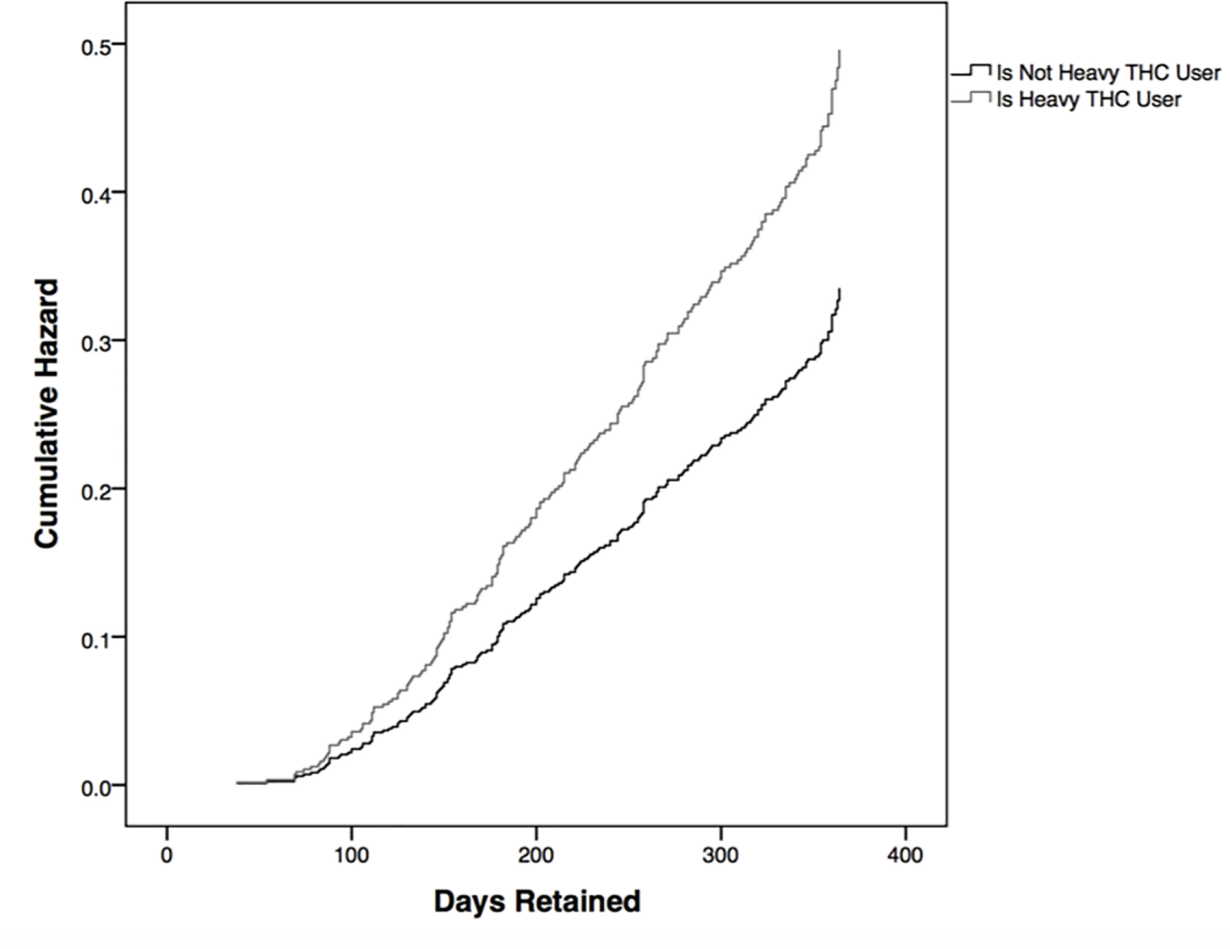
Likelihood of treatment dropout by proportion of cannabis-positive urine samples. A Cox proportional hazard analysis was used to characterize the time to treatment discontinuation between the patient groups. Heavy cannabis users were 48.1% more likely to drop out of treatment than non-heavy users [_a_HR = 1.481 (95% CI 1.134–1.933)].

Of the 644 patients, 388 (60.2%) had cannabis-positive urine samples less than 75% of the time, and 256 (39.8%) patients had cannabis-positive urine samples 75% or more of the time. A Cox proportional hazard analysis was used to characterize the time to treatment discontinuation across the two patient groups. Patients with 75% or more of urines cannabis-positive were 48.1% more likely to drop out of treatment than those with less than 75% of urines be cannabis-positive [_a_HR = 1.481 (95% CI 1.134–1.933)] (Fig 2).

### Cannabis use and geography

Although the proportion of Northern patients was higher in the baseline positive group than in the baseline negative group (Table 1), this difference was not significant. Therefore, there were no significant differences with respect to Northern/Southern location and baseline cannabis use in this patient population.

### Cannabis use and sex differences

When exploring the impact of cannabis use on retention by gender, different patterns arose. Female patients who were baseline positive for THC were 76.3% more likely to drop out of treatment than female non-baseline patients [_a_HR = 1.763 (95% CI 1.091–2.847)]. However, male patients who were baseline positive were no more likely to drop out of treatment than male non-baseline patients. Alternatively, female patients who were heavy cannabis users were no more likely to drop out of treatment than female non-heavy users; however, male patients who were heavy cannabis users were 45.0% more likely to drop out of treatment than male non-heavy users [_a_HR = 1.450 (95% CI 1.049–2.003)].

## Discussion

The findings of this study add some clarity to the impact of cannabis use in OAT, but also raise a variety of questions. Overall, patients who exhibit cannabis use upon entering OAT are more likely to be heavy cannabis users throughout treatment, and are also at increased risk of premature dropout. Given that approximately half of our patient sample was considered a baseline user, the implications of cannabis use need to be better understood since these patients are at increased risk of premature dropout. The same is also true for those patients who are heavy cannabis users—defined as having 75% or more of their urines THC positive—a group that should be carefully monitored throughout treatment. Patients with heavy cannabis use may be considered complex patients with multiple substance use disorders, therefore making their course of treatment more challenging and ultimately increasing their likelihood of premature dropout. It may also be the case that heavy users consume cannabis for the purpose of self-medication. In fact, the Canadian Alcohol and Drug Use Survey found that 24% of cannabis users report using cannabis for medical purposes[2]. Therefore, patients with heavy cannabis use may be attempting to self-medicate for anxiety, post-traumatic stress disorder, or other mental health disorders, conditions that may also make patients more likely to drop out of treatment.

Our findings beg a variety of questions, including why baseline cannabis use in women impacts treatment retention, while the equivalent use in males does not. There are some known differences in drug use patterns amongst males and females that may provide insight as to this discrepancy. Firstly, studies have found that 60% of males and 44% of females in OAT self-report cannabis use[30]. In this study, 62.8% of males were considered baseline users, but only 37.2% of females met the same criteria. Despite the difference in numbers, it appears that cannabis use is more prevalent in the male population; however, there may be added complexities for those females who do engage in cannabis use. Our findings suggest that when differentiating between males and females, only females are negatively impacted by baseline cannabis use. Given that females experience more significant opioid cravings when entering treatment[31] and are more likely to experience psychiatric comorbidity and illness than males[16, 32, 33], it may be that females generally have a more complex clinical course. Therefore, it may be the case that women use cannabis to self-medicate—whether for pain or anxiety—and that these conditions make them more likely to drop out of treatment in the first place, unrelated to their cannabis use. On the other hand, when evaluating the impact of heavy cannabis use on treatment retention by gender, it appears that only males are negatively impacted. Studies have found that males are at increased risk of developing cannabis use disorder[34]; therefore, of all heavy cannabis users, it may be that males are more likely to develop cannabis use disorder, adding to their clinical complexity and increasing their likelihood of premature dropout.

Previous research by our group has shown that patients in Northern Ontario have higher treatment retention than those in Southern Ontario [35]. This regional difference in treatment retention cannot be explained on the basis of the impact of cannabis use, as the patients in Northern regions of the province had higher rates of baseline cannabis use in this study (Table 1).

The findings of this study contribute to the body of research that is currently conflicted with regards to the impact of cannabis use in OAT[23–26]. The inconsistent findings in the current literature may be attributed to differing definitions of cannabis use, differing sensitivities of urine drug screen analysis, or perhaps unique demographics (e.g. rural and Northern patients). This particular study has some limitations that warrant discussion. Firstly, the length of time that THC remains detectable in urine varies by person and extent of cannabis use, which may lead to inconsistencies with the accuracy of urine drug screen analysis. Secondly, due to the nature of THC remaining in the system longer than other drugs, the classification of heavy cannabis use may be misleading as it could be possible that occasional users were categorized as heavy users based on the timing of their urine drug screening. Thirdly, due to the retrospective nature of the data, we were unable to determine various drug use details, including the cannabis use history or the amount of cannabis used. Lastly, if a patient dropped out of treatment, we were unable to determine whether they transitioned to a non-OATC clinic, terminated all OAT, were incarcerated, hospitalized, or died. This study also has many strengths, one of which being that it did not rely on self-reported data.

The findings from this study have broad implications. While studies have shown a potential for cannabis legalization to be a positive change for the population as a whole, there may be unique implications for those patients receiving OAT. Physicians should carefully monitor those patients who enter treatment cannabis-positive, as this may be a marker for a more complex clinical course, including heavy cannabis use and premature dropout. These patients should receive education surrounding the potential harms of cannabis use, including worsened OAT outcomes. Of particular concern should be female baseline cannabis users and males who demonstrate heavy cannabis use, both of which are at particularly increased risk of premature treatment dropout.
